# A Novel Minidumbbell DNA-Based Sensor for Silver Ion Detection

**DOI:** 10.3390/bios13030358

**Published:** 2023-03-08

**Authors:** Jiacheng Zhang, Yuan Liu, Zhenzhen Yan, Yue Wang, Pei Guo

**Affiliations:** 1 School of Biology and Biological Engineering, South China University of Technology, Guangzhou 510006, China; 2 Zhejiang Cancer Hospital, Institute of Basic Medicine and Cancer (IBMC), Chinese Academy of Sciences, Hangzhou 310022, China; 3 South China Advanced Institute for Soft Matter Science and Technology, School of Emergent Soft Matter, Guangdong Provincial Key Laboratory of Functional and Intelligent Hybrid Materials and Devices, South China University of Technology, Guangzhou 510640, China; 4 Institute of Molecular Medicine, Renji Hospital, School of Medicine, Shanghai Jiao Tong University, Shanghai 200127, China

**Keywords:** DNA sensor, silver ion detection, minidumbbell, non-B DNA, C·C mismatch

## Abstract

Silver ion (Ag^+^) is one of the most common heavy metal ions that cause environmental pollution and affect human health, and therefore, its detection is of great importance in the field of analytical chemistry. Here, we report an 8-nucleotide (nt) minidumbbell DNA-based sensor (*M-DNA*) for Ag^+^ detection. The minidumbbell contained a unique reverse wobble C·C mispair in the minor groove, which served as the binding site for Ag^+^. The *M-DNA* sensor could achieve a detection limit of 2.1 nM and sense Ag^+^ in real environmental samples with high accuracy. More importantly, the *M-DNA* sensor exhibited advantages of fast kinetics and easy operation owing to the usage of an ultrashort oligonucleotide. The minidumbbell represents a new and minimal non-B DNA structural motif for Ag^+^ sensing, allowing for the further development of on-site environmental Ag^+^ detection devices.

## 1. Introduction

Silver ion (Ag^+^) has been widely used as an antiseptic in cosmetics, building materials, and medical products owing to its antibacterial properties [1,2,3,4]. However, overuse of Ag^+^ inevitably leads to environmental pollution. Human exposure to Ag^+^ pollution mainly comes from the release of airborne silver nanoparticles and natural water contaminated by industrial sources [5,6]. The tolerable concentration of Ag^+^ in drinking water is ~927 nM as recommended by the World Health Organization [7]. Excessive Ag^+^ ingestion can cause certain serious health consequences, such as respiratory system injury, organ failure, and even cancer [6,8,9,10,11]. Various methods have been developed for detecting low concentrations of Ag^+^ in environmental samples and drinking water sources. At present, Ag^+^ detection is mainly carried out by conventional analytical methods such as inductively coupled plasma mass spectrometry [12], optical emission spectrometry [13], atomic absorption spectrometry [14,15], and laser ablation microwave plasma torch optical emission spectrometry [16]. These conventional methods are sensitive and selective, but they rely on expensive instruments and intensive labor.

In recent years, nucleic acid molecules have gained prominence in the fields of sensing and material science because of their programmability and predictability by forming complementary base pairs [17]. DNA molecules have been used to design sensors for detecting metal ions such as Ag^+^, UO_2_^2+^, Cu^2+^, Ca^2+^, Mg^2+^, Hg^2+^, and Pb^2+^ [18,19,20,21,22,23,24,25,26]. In general, there are mainly two DNA-based strategies for Ag^+^ detection. The first strategy utilizes an Ag^+^-dependent DNAzyme that can irreversibly cleave an RNA or DNA substrate in the presence of Ag^+^ [22]. The second strategy is based on the well-established knowledge that Ag^+^ binds to cytosine (C) at the N3 site to coordinate and stabilize a C·C mismatch [27,28]. Ag^+^ can induce the formation of DNA i-motif or hairpin structures that contain C·C mismatch(es), thus giving reporting signals upon DNA conformational change [26,29,30,31,32,33]. Moreover, the duplex or hairpin-forming strands can also be assembled onto nanomaterials for signal amplification [34,35,36,37,38]. The second strategy can achieve a low detection limit, but the reported ones generally used relatively long oligonucleotides, which might make the Ag^+^-induced DNA conformational change slow. For instance, a DNA sensor based on a 20-nucleotide (nt) hairpin required an incubation time of at least 10 min for Ag^+^ detection. Therefore, a DNA sensor using a short oligonucleotide is expected to have advantages of fast response, easy operation, and probably anti-interference capability in a complex environment, which allow for the further development of on-site environmental detection devices [33,39].

**Figure 1:**
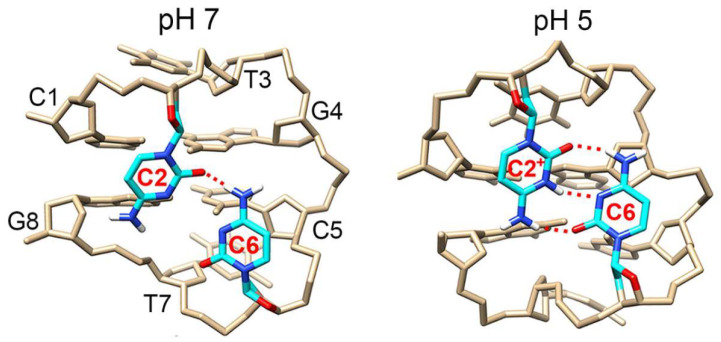
The averaged solution nuclear magnetic resonance (NMR) structure of the CCTG MDB at pH 7 (PDB ID: 5GWL) and pH 5 (PDB ID: 7D0Z). C2 and C6 formed predominantly a one-hydrogen-bond mispair at pH 7, whereas they formed a stable three-hydrogen-bond mispair at pH 5 with C2 being protonated.

Minidumbbell (MDB) is a type of non-B DNA structure formed by 8–10-nt sequences [40,41]. The MDB structure was initially found to form in CCTG tetranucleotide repeats, which are associated with the neurodegenerative disease of myotonic dystrophy type 2 [40,41]. The CCTG MDB is simply composed of two repeats, i.e., 5’-CCTG CCTG-3’, and each repeat folds into a type II tetraloop. The C1-G4 and C5-G8 adopt Watson-Crick loop-closing base pairs; C2 and C6 fold into the minor groove, whereas T3 and T7 stack on the C1-G4 and C5-G8, respectively (Figure 1) [40]. One of the most interesting features of this MDB is that the two minor groove residues formed a unique reverse wobble C2·C6 mispair containing one/two hydrogen bond(s) or Na^+^-mediated electrostatic interactions at neural pH [42], or a C2^+^·C6 mispair containing three hydrogen bonds with C2 being protonated at acidic pH (Figure 1) [43]. Upon lowering the pH from 7 to 5, the melting temperature (*T_m_*) of the CCTG MDB was increased from ~20 °C to 46 °C [43]. Apart from pH, we wondered if Ag^+^ could coordinate the C2·C6 mispair to stabilize the MDB and then induce a DNA conformational change for Ag^+^ sensing. Here we report a novel and minimal DNA sensor, based on the CCTG MDB, for Ag^+^ detection with high sensitivity and fast kinetics.

## 2. Materials and Methods

### 2.1. DNA Sequence Design and Sample Preparation

Our designed *M-DNA* sensor was a duplex formed by the CCTG MDB strand (5′-CCTG CCTG-3′) and its complementary strand (5′-CAGG CAGG-3′), which were named *CCTG_2_* and *CAGG_2_*, respectively. As a control, a self-complementary 8-bp duplex formed by 5′-GCAGCTGC-3′ was used. The high-performance liquid chromatography (HPLC)-purified DNA samples were purchased from Sangon Biotech (Shanghai, China), and they were further purified in our laboratory using diethylaminoethyl sephacel anion exchange column chromatography and Amicon Ultra-4 centrifugal filter devices. The ultra-violet (UV) absorbance at 260 nm was measured for DNA quantitation.

### 2.2. Preparation of SYBR Green I (SGI) and Metal Ion Stock Solutions

SGI (10,000×) was purchased from Beijing Solarbio Science and Technology Co., Ltd. (Beijing, China) and diluted using DMSO to a final concentration of 100× or 10× as the stock solution. It is noted that SGI 1× was equivalent to a concentration of 1.96 μM. The analytical-grade AgNO_3_, KCl, LiCl, CaCl_2_, MgCl_2_, MnCl_2_, CoCl_2_, CuSO_4_, BaCl_2_, and NiSO_4_ were purchased from Sinopharm Chemical Reagent Co., Ltd. (Beijing, China) and dissolved using DI water to a final concentration of 50 μM as the stock solutions.

### 2.3. NMR Experiments

To monitor the binding of Ag^+^ to the CCTG MDB, NMR experiments were performed using a Bruker AVANCE NEO 400 MHz spectrometer. One-dimensional (1D) 1H NMR experiments were conducted at 25 °C using the excitation sculpting pulse sequence to suppress the water signal.

### 2.4. Circular Dichroism (CD) Experiments

CD experiments were performed using a Chirascan V100 CD spectrometer with a bandwidth of 1 nm at room temperature, unless otherwise specified. The CD samples (~100 µL) were placed in a cuvette of 0.5 mm path length, and the CD spectra were collected from 200 to 350 nm with a step size of 1 nm. For each sample, three sets of scans were acquired, and an average value was taken. CD spectra were background-corrected using the corresponding buffer solution.

### 2.5. Fluorescence Experiments

Fluorescence experiments, except for the kinetic study of Ag^+^ sensing, were performed using a Shimadzu RF-6000 spectrometer at room temperature. The fluorescence samples (~2 mL) were placed in a 10 mm four-sided glazed quartz cuvette, and the fluorescence spectra were collected from 512 to 650 nm with a step size of 1 nm. Fluorescence intensity was recorded at 520 nm with an excitation wavelength of 492 nm. The excitation and emission band widths were 5 nm. For a kinetic study of Ag^+^ sensing, fluorescence experiments were performed using an Edinburgh FLS1000 photoluminescence spectrometer at room temperature. The sample containing a DNA sensor in the absence of Ag^+^ (~2.5 mL) was first placed in a 10 mm four-sided glazed quartz cuvette, and the fluorescence intensity at 520 nm was recorded from 0 to 180 s with a step time of 2 s. Ag^+^ was then added to this sample, and the fluorescence intensity was immediately recorded from 0 to 180 s with a step time of 2 s. The excitation and emission band widths were 2 nm.

The detailed sample conditions for NMR, CD, and fluorescence experiments are stated in the figure legends.

## 3. Results

### 3.1. Ag^+^ Induces a Conformational Change from Duplex to MDB

**Figure 2:**
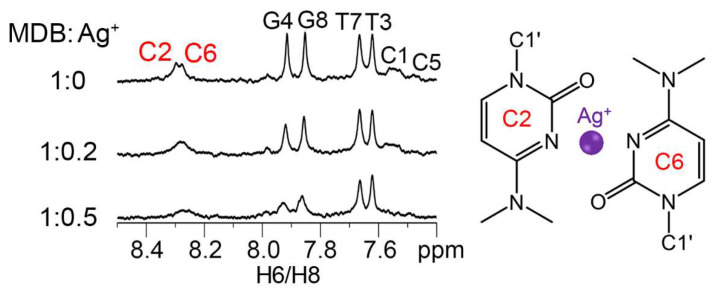
NMR spectra of 0.1 mM CCTG MDB in 1 mM sodium cacodylate (pH 6), 90% H_2_O/10% D_2_O, with various Ag^+^ concentrations at 25 °C. Peak broadenings of C2 H6 and C6 H6 in the presence of Ag^+^ suggest that Ag^+^ is bound to C2·C6 mispair.

One-dimensional (1D) ^1^H NMR experiments were first performed to investigate if Ag^+^ could bind to C2·C6 mispair of the CCTG MDB. It showed that upon adding Ag^+^ to the CCTG MDB, the H6 proton signals of C2 and C6 became broadened while those of T3 and T7 remained sharp and almost unchanged, suggesting that Ag^+^ bound to the C2·C6 mispair (Figure 2). Besides, C1 H6, G4 H8, C5 H6, and G8 H8 peaks were also found to be broadened, as it has been reported that Ag^+^ could also bind to C-G base pairs [44].

**Figure 3:**
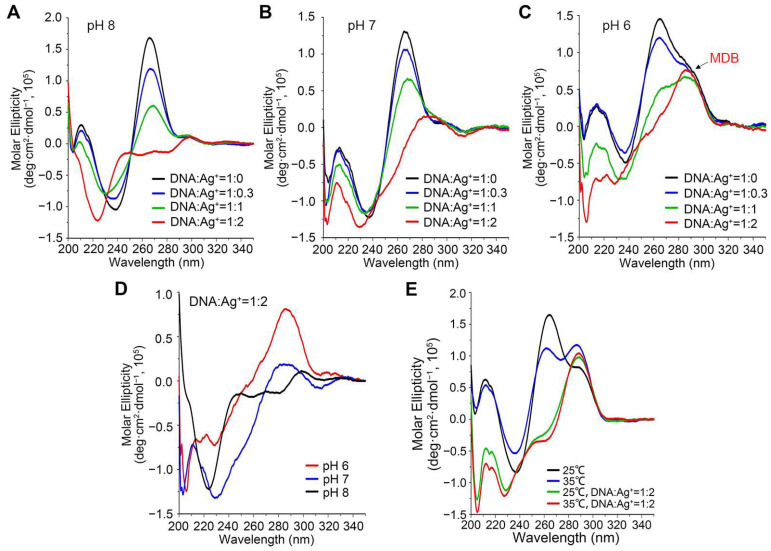
CD spectra of 15 μM *CCTG_2_* and *CAGG_2_* with 0, 5, 15, and 30 μM Ag^+^ in 10 mM NaPi at pH 8 (**A**), pH 7 (**B**), and pH 6 (**C**). (**D**) CD spectra of 15 μM *CCTG_2_* and *CAGG_2_* in 30 μM Ag^+^ at pH 8, 7, and 6 at 25 °C. (**E**) CD spectra of 15 μM *CCTG_2_* and *CAGG_2_* without Ag^+^ and with 30 μM Ag^+^ (pH 6) at 25 °C and 35 °C. Absorbance at 290 nm is characteristic of the free CCTG MDB.

We then tested if Ag^+^ could promote MDB formation to induce a DNA conformational change, which is the prerequisite of most DNA sensors. For this aim, we prepared a DNA duplex formed by the CCTG MDB strand (5′-CCTGCCTG-3′), namely *CCTG_2_*, and its complementary strand (5′-CAGGCAGG-3′), namely *CAGG_2_*, at pH 8/7/6 and collected CD spectra to monitor DNA conformational change upon Ag^+^ titration at 25 °C. These two strands formed a duplex in the absence of Ag^+^, as indicated by a positive CD band at 265 nm (Figure 3A–C, black lines) [45]. Upon adding Ag^+^ to the duplex, a new major band at 290 nm was observed at pH 6, but not obvious at pH 7 and 8, when the DNA:Ag^+^ ratio was 1:2 (Figure 3A–D, red lines). The CD band at 290 nm was characteristic of the CCTG MDB [46], suggesting that Ag^+^ efficiently induced a conformational change from the duplex to the MDB at pH 6. Notably, the DNA:Ag^+^ ratio of 1:2 showed the maximum population of Ag^+^-induced MDB (Figure 3C). This may because Ag^+^ is also non-selectively bound to C-G base pairs in the MDB (Figure 2), and thus more Ag^+^ is required to promote MDB formation.

We did not further lower the pH as previous work has demonstrated that the CCTG MDB completely dissociated from the duplex owing to its much higher thermodynamic stability than the duplex at pH 5 [43], therefore there would not be further conformational change upon adding Ag^+^. We also performed the Ag^+^ titration at 35 °C to examine if this system could function at an elevated temperature. However, the CD signal of MDB was observed without adding Ag^+^ (Figure 3E), which could be attributed to the relatively higher thermodynamic stability of MDB than duplex at 35 °C and pH 6. Zhang et al. have also reported that a higher temperature leads to partial melting of the initial DNA duplex and thus a lower sensitivity [47].

### 3.2. Design and Optimization of the CCTG MDB-Based DNA (M-DNA) Sensor

**Figure 4:**
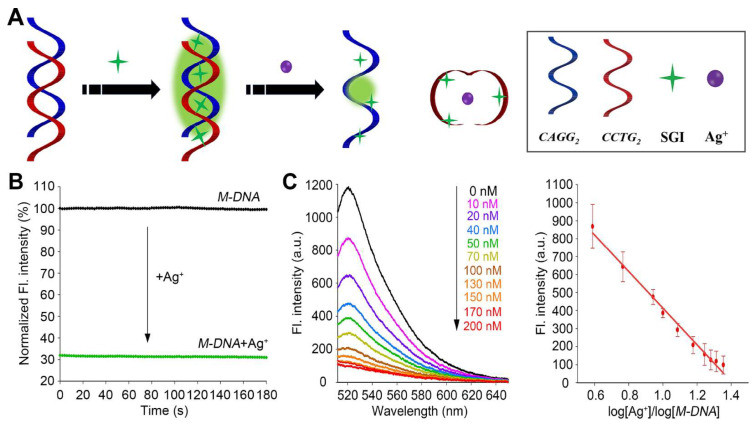
(**A**) Schematic of the *M-DNA* sensor for Ag^+^ detection. (**B**) Normalized fluorescence intensity at 520 nm as a function of time for the *M-DNA* sensor in the absence of Ag^+^ (black) and after adding 50 nM Ag^+^ (green). (**C**) Fluorescence spectra of the *M-DNA* upon titrating Ag^+^ ranging from 0 to 200 nM (**left**) and the fitting curve constructed using fluorescence intensity at 520 nm and log[Ag^+^]/log[*M-DNA*] (R^2^ = 0.99) (**right**). Error bars were standard deviations obtained from three replicative experiments.

Based on the Ag^+^-induced formation of CCTG MDB at pH 6 (Figure 3C,D), we designed the *M-DNA* sensor, which was simply composed of the 8-bp duplex formed by *CCTG_2_* and *CAGG_2_*. SYBR Green I (SGI) was used as a fluorescence reporter and it was expected to emit strong fluorescence when bound to the duplex in the absence of Ag^+^ while giving weak fluorescence when the duplex was converted to MDB in the presence of Ag^+^ (Figure 4A). To ensure SGI will not affect the DNA conformational change, CD spectra were collected without and with adding SGI, and the results showed that Ag^+^-induced conformational change still effectively occurred (Figure S1).

At pH 6, the *M-DNA* concentration and SGI:*M-DNA* ratio were further optimized. Two *M-DNA* concentrations (50 and 200 nM) and four SGI:*M-DNA* ratios (0.1:1, 0.5:1, 1:1, and 5:1) were tested to find the condition that would give the largest fluorescence change in response to Ag^+^. The DNA concentration and SGI:*M-DNA* ratio were finally optimized to be 50 nM and 1:1, respectively (Figure S2). Therefore, the *M-DNA* used for Ag^+^ sensing in the following experiments contained 50 nM *CCTG_2_*, 50 nM *CAGG_2_*, and 50 nM SGI in 10 mM NaPi at pH 6, unless otherwise specified.

To further verify whether the CCTG MDB played an important role in the *M-DNA* sensor for Ag^+^ detection, we also performed Ag^+^ titration on a controlled DNA (named *C-DNA*), which was an 8-bp self-complementary duplex. When the mixture of 50 nM *C-DNA* and 50 nM SGI in 10 mM NaPi at pH 6 was titrated with Ag^+^, there was only a little change in fluorescence intensity (Figure S3), suggesting that the CCTG MDB played an irreplaceable role in Ag^+^ sensing.

### 3.3. Kinetics, Sensitivity, and Selectivity of the M-DNA Sensor

One of the most interesting features of this *M-DNA* sensor is using an ultrashort 8-nt oligonucleotide, which is expected to undergo a much faster conformational change than longer i-motif and hairpin sequences [26,29,30,32]. Therefore, we also evaluated the kinetics of this *M-DNA* for Ag^+^ sensing. The fluorescence intensity (520 nm) of the *M-DNA* sensor without Ag^+^ was recorded from 0 to 180 s with a step time of 2 s. Ag^+^ was then added to the same sample, and the fluorescence intensity was immediately recorded from 0 to 180 s with a step time of 2 s. Figure 4B shows that immediately after adding Ag^+^, the fluorescence intensity drastically decreased and remained almost unchanged through the entire monitoring process for 180 s. Therefore, it is safe to conclude that the reaction was completed within the acquisition time for the first data point, i.e., 2 s. It was reported that the Ag^+^-triggered conformational change from a single-stranded DNA to a 21-nt i-motif was complected in ~15 s [26,29,30,32], therefore it is reasonable that the conformational change to an 8-nt MDB was much faster.

The *M-DNA* was then used to sense Ag^+^ at various concentrations ranging from 0 to 200 nM (Figure 4C). There was a good linear correlation between the fluorescence intensity and log[Ag^+^]/log[M-DNA]. Following the rule of three times the standard deviation over the blank response [48], the Ag^+^ detection limit was determined to be ~2.1 nM. As the tolerable level of Ag^+^ in drinking water is ~927 nM [7], the detection limit of the *M-DNA* sensor should be sufficient for detecting Ag^+^ in real samples containing Ag^+^.

**Figure 5:**
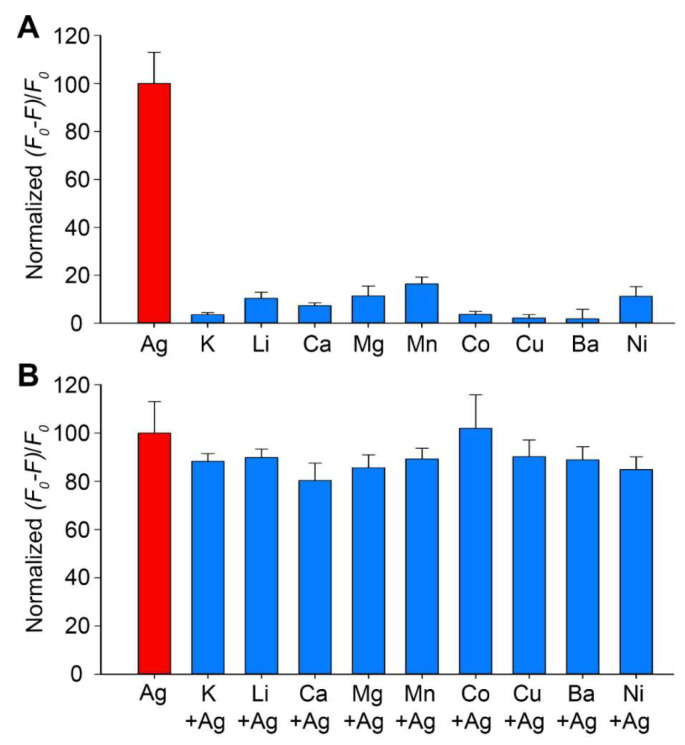
Fluorescence changes at 520 nm of the *M-DNA* in the presence of (**A**) 50 nM non-Ag^+^ metal ions (blue) and (**B**) 50 nM non-Ag^+^ metal ions plus adding 50 nM Ag^+^ (blue). The fluorescence change in the presence of only 50 nM Ag^+^ was shown as a reference (red). Error bars were standard deviations obtained from three replicative experiments. *F_0_*: initial fluorescence intensity in the absence of Ag^+^; *F*: fluorescence intensity after adding 50 nM AgNO_3_ or other metal ions.

The anti-interference capability of the *M-DNA* sensor for Ag^+^ detection in a complex environment was also evaluated. As the drinking water source may also contain other metal ions, we evaluated the fluorescence response of *M-DNA* to K^+^, Li^+^, Ca^2+^, Mg^2+^, Mn^2+^, Co^2+^, Cu^2+^, Ba^2+^, and Ni^2+^, and the result showed only tiny fluorescence changes upon adding these ions (Figure 5A). Furthermore, an additional experiment was also performed to examine if the *M-DNA* could detect Ag^+^ in the presence of these interfering metal ions. Upon adding 50 nM Ag^+^ to the solutions containing the respective interfering metal ions, the fluorescence change became significant and achieved a similar level to that of only 50 nM Ag^+^ (Figure 5B). Na^+^ was not included as an interference ion in this study because the buffering system contained 10 mM NaPi. Approximately 10 to 200 mM Na^+^ are also commonly used in buffering systems for many DNA-based sensors to neutralize the negatively charged phosphodiester backbones [26,29,30,31,34]. The concentrations of non-Ag^+^ ions vary in different water samples, e.g., few mM Na^+^ in most China river and lake basins [49] and hundreds mM Na^+^ in sea water [50]. The *M-DNA* sensor should be applicable for detecting Ag^+^ in common river and lake basins, and its performance may need to be further improved for sensing Ag^+^ in water samples containing high concentrations of interfering ions (e.g., sea waters).

### 3.4. Ag^+^ Detection in Tap Water and Lake Water Samples Using the M-DNA Sensor

To examine the performance of the *M-DNA* sensor for Ag^+^ detection in other water sources, we detected Ag^+^ in tap water samples and two different lake water samples. The local tap water and lake water samples were collected and boiled for 5 min to remove chlorine, and lake water samples were further filtered with a 0.22 µm membrane following the reported procedures in the literature [26]. The *M-DNA* sensor was prepared using the treated tap and lake water samples instead of laboratory DI water, and no Ag^+^ was detectable in these samples. We then added Ag^+^ with known concentrations to the *M-DNA* sensor and recorded the fluorescence intensity. The Ag^+^ concentration was calculated using the calibration curve shown in Figure 4C. The recovery ranged from 93.3% to 98.5% in tap water samples and 96.7% to 107.8% in lake water samples (Table 1), revealing a good accuracy of the *M-DNA* sensor for Ag^+^ detection in environmental water sources.

### 3.5. Discussions on DNA-Based Ag^+^ Sensors

As surveyed from the literature, DNA-based Ag^+^ sensors can be generally classified into three types: (i) mismatch-containing DNA functionalized with nanomaterials [34,35,36,37], (ii) mismatch-containing DNA only [26,29,30,32], and (iii) DNAzyme [22] (Table 2). The ensemble of mismatch-containing DNA and nanomaterials is an effective strategy to improve the detection limit by taking advantage of amplified local DNA concentration and interaction surfaces. Recently, Pal et al. have reported an electrochemical Ag^+^ sensor based on DNA hairpin-functionalized nanoflakes with a detection limit of 0.8 pM [38]. Comparing with the detection limits of other sensors using only mismatch-containing DNA (i-motifs and hairpins), detection limit of the *M-DNA* sensor was the lowest. In addition, the *M-DNA* sensor exhibited a response time of less than 2 s, which is kinetically much faster than those using i-motifs and hairpins (Table 2). However, the *M-DNA* sensor requires a controlled acidic pH to work, and this limitation may be further improved by chemical modification, such as cytosine methylation, to enhance the thermodynamic stability of the CCTG MDB. Overall, the *M-DNA* sensor uses an ultrashort oligonucleotide to achieve a high sensitivity and fast response for Ag^+^ detection.

## 4. Conclusions

In sum, we have designed a smart DNA sensor for Ag^+^ detection using a new form of non-B DNA, i.e., a minidumbbell, apart from the previously used hairpins and i-motifs. Owing to its small size, it shows fast response, high sensitivity, high selectivity, and good anti-interference capability for Ag^+^ sensing. The performance of this *M-DNA* sensor may be further improved by chemical modification to further enhance the thermodynamic stability of the CCTG MDB. A successful demonstration of this *M-DNA* sensor provides new insights into Ag^+^ detection, and paves the way for designing DNA-based tools to sense other metal ions and molecules.

## Figures and Tables

**Table 1:** Ag^+^ detection in tap and lake waters using the *M-DNA* sensor.

Water Source	*C_real_* (nM)	*C_cal_* (nM) ^a^	Recovery (%)
Tap water	45	42 ± 4	93.3
	90	86 ± 4	95.6
	130	128 ± 5	98.5
	150	143 ± 6	95.3
Lake water 1	45	46 ± 1	102.2
	90	97 ± 8	107.8
	130	133 ± 6	102.3
	150	152 ± 6	101.3
Lake water 2	45	46 ± 4	102.2
	90	87 ± 7	96.7
	130	133 ± 25	102.3
	150	155 ± 17	103.3

^a^ The standard deviations were obtained from three replicative experiments.

**Table 2:** Literature survey on DNA-based sensors for Ag^+^ detection.

DNA Sensor	DNA Length (nt)	Kinetics	Detection Limit	Ref.
DNA/graphene oxide	32	^b^	5 nM	[34]
DNA/silver nanoclusters	12	<1 min ^a^	10 nM	[35]
DNA/gold nanoparticle	27	^b^	3.5 nM	[36]
DNA/Fe3O4-gold nanoparticle	49	^b^	3.4 nM	[37]
DNA/nanoflakes	20	^c^	0.8 pM	[38]
DNAzyme	83	60 min ^a^	24.9 nM	[22]
DNA hairpin	32	5 min ^a^	59.9 nM	[26]
DNA hairpin	20	10 min ^a^	32 nM	[29]
DNA hairpin	32	30 min ^a^	4.3 nM	[30]
DNA i-motif	21	15 s ^a^	17 nM	[32]
DNA minidumbbell	8	<2 s ^a^	2.1 nM	This work

^a^ The kinetic data was derived from time-dependent fluorescence spectra. ^b^ There was no kinetic data available. ^c^ The kinetic data was derived from time-dependent electrochemical change.

## Data Availability

The data presented in this study are available in supplementary material.

## Supplementary Materials

The following supporting information can be downloaded at: https://www.mdpi.com/article/10.3390/bios13030358/s1, Figure S1: CD changes of *M-DNA* with SGI in 10 mM NaPi at pH 6 before and after adding Ag^+^; Figure S2: Fluorescence changes of various-concentration *M-DNA* in 10 mM NaPi at pH 6, with different SGI:*M-DNA* ratios before and after adding Ag^+^; Figure S3: Normalized fluorescence intensity at 520 nm of the *M-DNA* and *C-DNA* upon titrating various concentrations of Ag^+^.
