# Supplementary material for: A Novel Minidumbbell DNA-Based Sensor for Silver Ion Detection
Source: Biosensors (Basel). 2023 Mar 8;13(3):358. doi: 10.3390/bios13030358 (PMC10046540; doi:10.3390/bios13030358)
Supplement: Supplementary file 1 [file biosensors-13-00358-s001.zip › biosensors-2183870-supplementary.pdf]

biosensors

MDPI

Communication

A Novel Minidumbbell DNA-Based Sensor for Silver Ion Detection

Jiacheng Zhang [1,2,†], Yuan Liu [3,†], Zhenzhen Yan [1,2], Yue Wang [4,*] and Pei Guo [2,*]

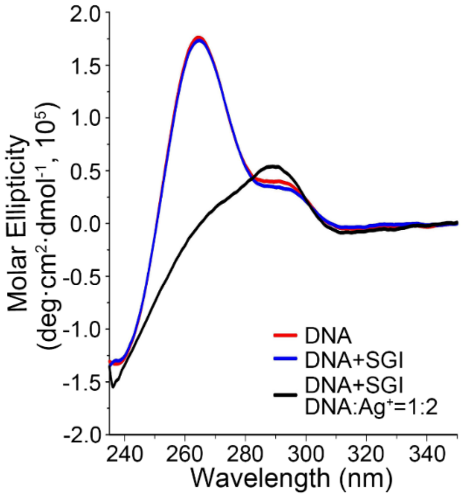

Figure S1. CD spectra of 15 μM *CCTG*$_2$ and *CAGG*$_2$ with 50 nM SGI in 10 mM NaPi at pH 6 before and after adding 30 μM Ag^+.

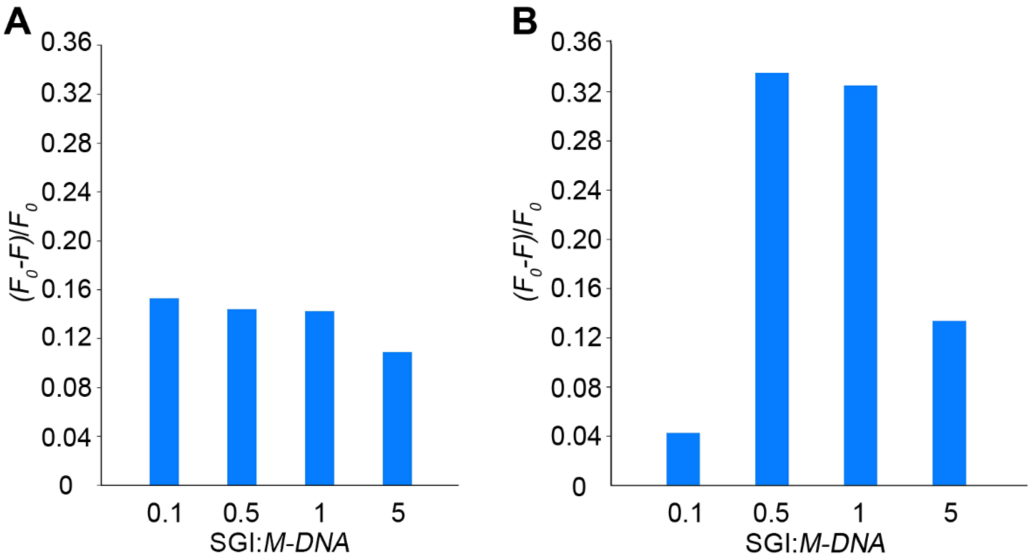

Figure S2. Fluorescence changes of (A) 200 nM *M-DNA* and (B) 50 nM *M-DNA* in 10 mM NaPi at pH 6 with different SGI:*M-DNA* ratios before and after adding Ag^+. F_0: initial fluorescence intensity at 520 nm in the absence of Ag^+, F: fluorescence intensity at 520 nm after adding 400 nM $AgNO_3$.

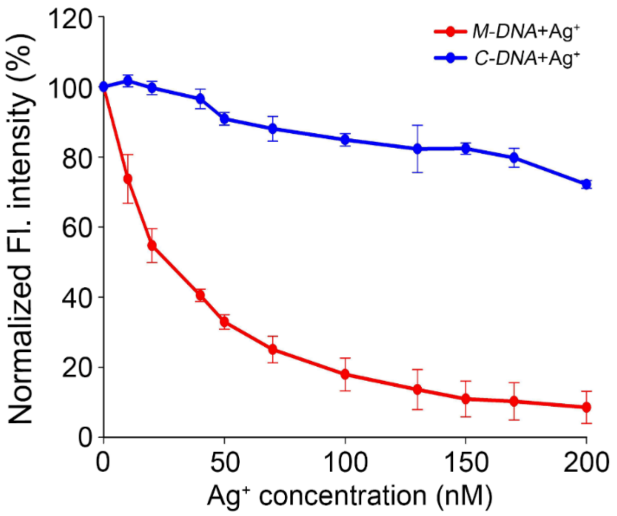

Figure S3. Normalized fluorescence intensity at 520 nm of the *M-DNA* (red) and *C-DNA* (blue) upon titrating various concentrations of Ag^+. Error bars were standard deviations obtained from three replicative experiments. Condition of sample for Ag^+ titration: 50 nM DNA and 50 nM SGI in 10 mM NaPi at pH 6.
